# The association between autism, camouflaging and anxiety with suicidal ideation in women

**DOI:** 10.3389/fpsyg.2025.1685845

**Published:** 2026-01-23

**Authors:** Sabela Conde-Pumpido-Zubizarreta, Sara Cruz, Marta Pozo-Rodríguez, José Javier Suárez-Rama, Ananta Díaz-Hernández, Angel Carracedo, María Tubío-Fungueiriño, Montse Fernández-Prieto

**Affiliations:** 1 Genomics and Bioinformatics Group, Center for Research in Molecular Medicine and Chronic Diseases (CiMUS), Universidade de Santiago de Compostela (USC), Santiago de Compostela, Spain; 2 Department of Education and Psychology, William James Center for Research, University of Aveiro, Aveiro, Portugal; 3 Genetics Group, Instituto de Investigación Sanitaria de Santiago (IDIS), Santiago de Compostela, Spain; 4 Fundación Pública Galega de Medicina Xenómica, Santiago de Compostela, Spain; 5 Fundación Pública Galega Instituto de Investigación Sanitaria de Santiago de Compostela (FIDIS), Santiago de Compostela, Spain; 6 Centro de Investigación Biomédica en Red de Enfermedades Raras, Instituto de Salud Carlos III, Madrid, Spain

**Keywords:** anxiety, autism, camouflaging, suicidal ideation, women

## Abstract

**Introduction:**

Camouflaging behaviors in women have been associated with mental health outcomes, like anxiety and suicidality but the mechanisms underlying these relationships remain unclear. This study aimed to examine the relationship between autism, camouflaging and anxiety with suicidal ideation and to investigate the possible mediating role of anxiety in the relationship between camouflaging and suicidal ideation in women.

**Methods:**

Four hundred and seventy-one women (72 autistic, aged 18–64 years, and 399 non-autistic, aged 18–66 years) participated in this study. The Camouflaging Autistic Traits Questionnaire (CAT-Q), the General Anxiety Disorder 7 (GAD-7) and item 9 of the Patient Health Questionnaire 9 (PHQ-9) were used to assess camouflaging, anxiety and suicidal ideation, respectively.

**Results:**

The results showed that autistic women scored higher than non-autistic women on all measures. In addition, anxiety, having a depression diagnosis and the assimilation strategy were significantly associated with suicidal ideation. Furthermore, anxiety mediated the relationship between autism diagnosis and camouflaging with suicidal ideation.

**Discussion:**

The results highlight the importance of considering anxiety in the relationship between camouflaging and suicidal ideation, particularly among autistic women, and to recognise it as a target for intervention approaches aimed at reducing the likelihood of suicidal ideation.

## 1 Introduction

Autism spectrum disorder (ASD) is a neurodevelopmental condition characterized by differences in communication and interaction with other people, and by restricted interests and repetitive behaviours that affect functioning in different areas of life (American Psychiatric Association, 2022). Autism is more often diagnosed in boys (Cruz et al., 2024), with possible gender differences in the phenotype of autism contributing to girls being diagnosed later or underdiagnosed (Loomes et al., 2017).

Indeed, a ‘female autism phenotype’ has been proposed, positing that the presentation of autistic women is qualitatively different from the typical presentation of men (Hull et al., 2017), contributing to the diagnostic imbalance between boys and girls (Cruz et al., 2024; Hull et al., 2020). Research still holds the hypothesis toward a male-centered vision of autism that lacks to consider internalizing symptoms, which are more commonly seen in autistic women, potentially leading to misdiagnosis (Beggiato et al., 2017; Cruz et al., 2024; Mao et al., 2024). Autistic women are also associated with increased camouflaging, i.e., the use of social strategies to decrease the visibility of their autistic characteristics to fit in social contexts (Hull et al., 2020). Thus, camouflaging is hypothesized as a contributor to women having higher mental health challenges (e.g., anxiety and depression), compared to non-autistic women with mental health conditions (Dubreucq and Dubreucq, 2021). Moreover, autistic women appear to be at more risk of suicidal ideation (i.e., thoughts of self-harm or of ending one’s life) and suicide history relative to men (Dubreucq J. et al., 2023; Dubreucq M. et al., 2023; Harmer et al., 2024; Newell et al., 2023).

However, more research is needed to understand the interplay between autism, camouflaging, anxiety, and suicidal ideation in women, as these associations remain understudied. In line with this, recent research has highlighted the importance of clarifying the relationship between camouflaging and suicidal thoughts and behaviours, with a particular focus on examining potential mediators of this relationship (Arqueros et al., 2025). Understanding factors associated with suicidal ideation is essential for the development of targeted intervention approaches to reduce suicidal thoughts or behaviours and improve quality of life in this population (Harmer et al., 2024).

### 1.1 Gender, mental health, and autism in women

Gender is a complex concept that involves psychosocial and cultural aspects and is influenced by social practices, norms, and expectations of others (World Health Organization, 2014). Gender has a significant impact on mental health, with women being particularly vulnerable to mental health problems, especially internalising disorders (e.g., anxiety or depression), in part due to exposure to social (e.g., discrimination), economic (e.g., poverty) and environmental (e.g., inequalities) stressors (Baños and Miragall, 2024; Seedat et al., 2009). Compared to men, women are at higher risk of suicidal ideation, with distinct gender-specific risk factors, such as mental illness or feelings of hopelessness, playing an important role (Shelef, 2021).

These gender-related aspects are also relevant when considering autism in women. Although women have similar core autistic traits to men, they are often misdiagnosed or underdiagnosed in part because their behavioural expression of autism, although similar to the underlying characteristics described in the diagnostic criteria, may differ from the traditional male expression, with less obvious social impairments (Belcher et al., 2023; Kopp and Gillberg, 1992). This may relate to the fact that women typically exhibit more social behaviours than autistic men (Dubreucq and Dubreucq, 2021), which may be explained by gendered social norms or expectations regarding women (Tubío-Fungueiriño et al., 2021). Women’s presentation of autism therefore differs from the typical male presentation in that autistic characteristics may be expressed differently from the traditional criteria. For example, compared to autistic men, autistic women have higher levels of social motivation (e.g., having friendships with others) (Head et al., 2014; Sedgewick et al., 2016), and differences in the profile of restricted and/or repetitive behaviours and focused interests, or their interests may be more in accordance with their age (i.e., more focused on relational aspects) (Allely, 2019).

The fact that autistic women seem to have more social communication/interaction abilities (described as narrow constructs, such as social attention), may be associated with their ability to camouflaging their autistic traits to fit into social environments (Wood-Downie et al., 2021). Camouflaging refers to using social strategies to minimise the visibility of autism or autistic traits to facilitate social integration and peer acceptance (Hull et al., 2020; Lai and Baron-Cohen, 2015; Tubío-Fungueiriño et al., 2021). Camouflaging strategies include assimilation, i.e., the adoption of observed attitudes and/or behaviours to fit into social situations; compensation, i.e., the performance of behaviours to overcome social difficulties associated with autistic traits; and masking, i.e., the concealment/hiding of autistic traits (Hull et al., 2020). Autistic girls identify and learn social behaviours from others (for example, facial expressions, or making eye contact) that are considered ‘appropriate’ behaviours, which are then reinforced by social expectations, increasing their expression (Lai et al., 2017).

However, camouflaging has important psychological consequences for autistic women, as it has been associated with higher anxiety and lower mental wellbeing (Conde-Pumpido Zubizarreta et al., 2025c; Khudiakova et al., 2024) and linked to an increased risk of suicidal ideation (Cassidy et al., 2020). These relationships are partly explained by the fact that efforts to suppress or hide autistic traits require significant cognitive and emotional resources, which may, in effect, contribute to increased levels of internalising disorders (i.e., anxiety and depression) (Hull et al., 2017).

### 1.2 Suicidal ideation, autism, camouflaging, and anxiety in women

Suicidal ideation refers to thinking about or planning to commit suicide and is closely associated with suicide attempts and deaths and is considered as a significant risk factor for future suicidal behaviours (Harmer et al., 2024). Compared to non-autistic individuals, autistic people are at greater risk, as rates of suicidal ideation in autistic adults are significantly higher than in the general population (Hirvikoski et al., 2020). Within autistic adults, women appear to be a particularly vulnerable group as they show elevated risk factors for suicidal ideation (i.e., thoughts and behaviours) compared with autistic men. One survey reported that 74.3% of autistic adults experienced suicidal ideation in the past year, with male sex linked to lower ideation (Moseley et al., 2022). A systematic review shows a pooled prevalence of 34.2% for suicidal ideation and supported previous evidence that suicidality is more prevalent in autistic women than men (Newell et al., 2023).

Recent research suggests that camouflaging is associated with increased suicidal ideation (Cassidy et al., 2023). A study of 58 adult women, who scored high on broad autistic traits found that camouflaging moderately predicted psychological distress and functional challenges (Beck et al., 2020). Another study of 160 undergraduate students (86.9% of whom were women), showed that camouflaging autistic traits was associated with a higher risk of experiencing thwarted belonging and lifetime suicidality (Cassidy et al., 2020).

However, the mechanism linking camouflaging and suicidal ideation is not yet clear. One possible explanation for this association is that camouflaging is strongly associated with greater symptoms of anxiety (Hull et al., 2021; Khudiakova et al., 2024), which in turn are strongly associated with increased suicidal ideation (Doering et al., 2024). Thus, the relationship between camouflaging and suicidal ideation may be mediated by anxiety. Camouflaging is often reported to be exhausting and stressful (Conde-Pumpido Zubizarreta et al., 2025a; Hull et al., 2017; Tierney et al., 2016), which can lead to feelings of being misunderstood or overlooked (Cage et al., 2018). Camouflaging is related to autistic burnout, as is shown by Benatov et al. (2025) that found that that autistic burnout may be a partial mediator between self-reported camouflaging behaviours and depression using the Patient Health Questionnaire (PHQ-9, Kroenke et al., 2001).

As autistic women are more likely than autistic men to use camouflaging strategies (Hull et al., 2017; Milner et al., 2023), and as they report higher rates of anxiety (Somerville et al., 2024), it is possible that autistic women are at increased risk of higher levels of anxiety and suicidal ideation, which may partly explain the increased psychological distress observed in this group. It is therefore important to clarify the mediating role of anxiety in the relationship between camouflaging and suicidal ideation in women. Clarifying these relationships will help to inform intervention programmes and gender-sensitive approaches.

### 1.3 Current study

Given that women often report higher rates of anxiety and depression, and that autistic women are at greater risk for mental health problems and more likely to use camouflaging, it is important to clarify the relationships between autism, camouflaging, anxiety and suicidal ideation in autistic and non-autistic women. The aim of this study is twofold: (i) to examine the contribution of autism diagnosis, camouflaging and anxiety to suicidal ideation, and (ii) to investigate the mediating role of anxiety in the relationship between camouflaging and suicidal ideation in women. Therefore, the present study includes both autistic and non-autistic women as a comparison group because we hypothesise that autistic women will report higher levels of camouflaging, anxiety, and suicidal ideation than non-autistic women. Furthermore, we expect that a diagnosis of autism, camouflaging and anxiety will be significantly and positively associated with suicidal ideation. We also expect anxiety to significantly mediate the association between camouflaging and suicidal ideation in autistic women.

## 2 Methods

### 2.1 Participants

A total of 471 women, 461 Spanish and 10 of other nationalities but living in Spain, participated in this study. Of these, 72 were autistic, aged 18–64 years (*M* = 36.06; SD = 10.79), and 399 were non-autistic, aged 18–66 years (*M* = 34.14; SD = 11.08). Table 1 provides detailed information on the participants’ level of education and co-occurring mental conditions.

**Table 1:** Level of education and self-reported clinical conditions for the autistic and non-autistic groups.

Educational level/clinical conditions	Autistic		Non-autistic	
	*N*	%	*N*	%
Level of education				
No studies	–	–	1	0.3
Elementary education	–	–	2	0.5
Secondary education	6	8.3	33	8.3
Degree	46	63.9	264	66.2
PhD	3	4.2	56	14
Higher vocational training	5	6.9	15	3.8
Vocational training	12	16.7	28	7
Self-reported clinical conditions				
High abilities	23	31.9	17	4.3
Attention deficit/hyperactivity disorder	17	23.6	15	3.8
Developmental coordination disorder	1	1.4	1	0.3
Specific learning disorder	6	8.3	5	1.3
Epilepsy	0	-	1	0.3
Bipolar disorder	1	1.4	0	–
Eating disorder	9	12.5	24	6
Anxiety	39	54.2	76	19
Depression	24	33.3	49	12.3
Sleeping disorder	15	20.8	18	4.5
Other	11	15.3	9	2.3

Inclusion criteria for both groups were age 18 years or older, Spanish nationality or residence in Spain, and capacity to consent and complete the self-report measures. For the autistic group, inclusion criteria also included having a formal diagnosis of autism (conducted by a health professional, such as psychiatrist or psychologist). Of note, the mean age of diagnosis of the participants was 32.88 years (age range = 6–58), with three women (4.2%) diagnosed before the age of 10, five (6.9%) between the ages of 13 and 19, 21 (29.2%) between the ages of 20 and 29, 22 (30.5%) between the ages of 30 and 39, and 21 (29.2%) between the ages of 40 and 58. Those who were suspected of having an ASD diagnosis but had not received a formal diagnosis were not included in this study.

### 2.2 Measures of dimensions assessed

#### 2.2.1 Sociodemographic information

Participants completed a sociodemographic questionnaire to collect information on gender, age, nationality, level of education, mental health (i.e., self-reported clinical conditions), and diagnosis of autism.

#### 2.2.2 Suicidal ideation

To assess suicidal ideation, we used the item 9 (‘Thoughts that you would be better off dead, or of hurting yourself’) of the Patient Health Questionnaire 9 (PHQ-9), Spanish version (Diez-Quevedo et al., 2001). This is a clinically validated screening tool used to assess and monitor the severity of depression based on the Diagnostic and Statistical Manual of Mental Disorders 4th edition (DSM-IV, American Psychiatric Association, 1994) criteria. It consists of nine items (e.g., ‘Feeling down, depressed, or hopeless’), answered on a Likert scale from 0 (‘not at all’) to 3 (‘almost every day’), which ask respondents how often they have been ‘bothered by any of the following problems’ in the last two weeks. The items cover sleep, energy, appetite and other possible symptoms of depression. The total score is calculated by adding up all the items and indicates how often a person experiences these feelings, with higher scores reflecting more severe symptoms. The PHQ-9 total score showed strong internal consistency and convergent validity in autistic and non-autistic adult samples (Arnold et al., 2020). Additionally, other studies find item 9 as a valid measure of suicidal ideation in autistic and non-autistic participants (Hedley et al., 2018; Simon et al., 2013).

In this study, we considered a score of 0 (‘not at all’) on item 9 as “absence of suicidal ideation,” and a score of 1 (‘several days’), 2 (‘more than half of the days’), or 3 (‘nearly every day’) as “presence of suicidal ideation.”

#### 2.2.3 Anxiety

To assess anxiety symptoms, we used the General Anxiety Disorder 7 (GAD-7), Spanish version (García-Campayo et al., 2010). GAD-7 is a widely used instrument that measures the severity of generalised anxiety disorder. It consists of seven items that measure the frequency of various anxiety symptoms over the past two weeks (e.g., ‘Worrying too much about different things’). Respondents answer each item on a Likert scale from 0 (‘not at all’) to 3 (‘nearly every day’).

The GAD-7 total score is obtained by summing up all the items and this value was used in the analysis. Higher scores indicate greater severity of anxiety symptomatology. Excellent internal consistency results were observed (*α* = 0.92).

#### 2.2.4 Camouflaging

To assess camouflaging behaviours, we used the Camouflaging Autistic Traits Questionnaire, Spanish version (CAT-Q-ES) (Conde-Pumpido Zubizarreta et al., 2025b). The CAT-Q-ES is a self-report measure that assesses social camouflaging behaviours. This questionnaire consists of 25 items (e.g., ‘When I am interacting with someone, I deliberately copy their body language or facial expressions), rated on a Likert scale from 1 (‘strongly disagree’) to 7 (‘strongly agree’). Three subscales – compensation (i.e., imitating social behaviours), masking (i.e., suppressing natural reactions) and assimilation (i.e., using coping strategies to manage social interactions) – and a total score are obtained. Higher scores indicate a higher level of camouflaging. Excellent internal consistency results were observed for the total score (α = 0.95), and for the compensation (α = 0.93) and assimilation (α = 0.92) subscales. The masking subscale showed acceptable internal consistency (α = 0.79).

In this study, the subscales and total scores were used to test for group differences. Only the three subscales were used in the regression analysis, as they are highly correlated with the CAT-Q-ES total score, and the CAT-Q-ES total score was used in the mediation analysis.

### 2.3 Procedure

This study is part of a broader project aimed at validating the CAT-Q in the Spanish population (accepted for publication). Data was collected and managed using REDCap (Research Electronic Data Capture) tools hosted at Fundación Pública Galega de Medicina Xenómica (Harris et al., 2009; Harris et al., 2019). REDCap is a secure, web-based software platform designed to support data capture for research studies, providing (1) an intuitive interface for validated data capture; (2) audit trails for tracking data manipulation and export procedures; (3) automated export procedures for seamless data downloads to common statistical packages; and (4) procedures for data integration and interoperability with external sources.

The research team created a survey on REDCap that was distributed through internal dissemination within autism associations, institutions, federations and clinical units, as well as autism-related research networks and social media. The survey was launched in February 2023, and the last response was received in March 2024. A total of 1,292 people participated. Of these, 709 surveys were fully competed; however, 19 of these participants had only completed the parental version of the CAT-Q-ES and were therefore excluded from this study. Although the survey requested that participants answer all questions, it was possible to proceed without responding.

The primary section of the survey provided information about the study and included an informed consent form. In this section, participants were asked about their clinical diagnosis of autism and how it was obtained (e.g., diagnosis performed by a psychologist). Autistic participants were also asked about the formal clinical diagnosis of other comorbidities associated with autism (for example, other neurodevelopmental conditions, bipolar disorder, or anxiety/depressive conditions). Non-autistic participants were asked if they had any other neurodevelopmental and/or psychiatric diagnoses.

After completing the sociodemographic questionnaire, participants proceeded to complete the other questionnaires (PHQ-9, GAD-7 and CAT-Q-ES). The completion of the survey lasted approximately 15 to 20 min.

### 2.4 Statistical analyses

Statistical analyses were conducted using *jamovi* software (version 2.7, 2025) (The Jamovi Project, 2025).

As the assumption of normality was not met (Shapiro-Wilks test < 0.05 for all variables), we conducted non-parametric tests. Following the recommendations of Fife-Schaw (2006), we also computed the equivalent parametric tests, as parametric methods can remain reasonably robust with non-normal continuous data when sample size guidelines are met (e.g., two groups with more than 15 participants; Banga and Fox, 2013). Because the pattern of results was consistent across both approaches, we report the parametric test results for ease of interpretation and comparability, noting that these tests are generally robust even under moderate violations of normality.

Descriptive statistics were computed for all the measures included in this study (item 9 of the PHQ-9, GAD-7 total score), and CAT-Q-ES questionnaire – compensation, masking, and assimilation subscales – and CAT-Q-ES total score. Then, an Independent-Samples T-Test was performed to test group differences regarding all dimensions, except the Item-9 of the PHQ, in which group differences were tested using Chi-Square (χ^2^) test.

A logistic regression analysis was performed to examine the associations between anxiety (GAD-7), camouflaging (CAT-Q-ES compensation, masking, and assimilation subscales; the total score was not included give the high correlation with the subscales) and age on suicidal ideation, controlling for autism diagnosis and having and anxiety and/or depression diagnosis.

A mediation analysis was conducted to test the mediating role of anxiety in the relationship between camouflaging and suicidal ideation. Standard errors were calculated using nonparametric resampling via the percentile bootstrapping method, and missing data were treated using the FIML method (Dong and Peng, 2013). A path model in general linear model (GLM) mediation model (using the jAMM module for mediation models) with Maxiumum Likelihood Estimation was used to test the hypothesis of anxiety (i.e., GAD-7 total score) in the role of mediating the relationship between camouflaging (i.e., CAT-Q-ES total score) and suicidal ideation (i.e., item 9), controlling for autism and depression diagnosis (i.e., autistic or non-autistic).

## 3 Results

### 3.1 Descriptive and group difference analyses

Regarding suicidal ideation, and considering all participants, the response distribution to the item 9 of the PHQ was as follows: 0 = 318 (68%), 1 = 81 (17%), 2 = 34 (7%), 3 = 38 (8%). The skewness was 1.58 (standard error = 0.11) and the kurtosis was 1.27 (standard error = 0.22), indicating a positively skewed distribution consistent with less frequent suicidal ideation. When dichotomized (i.e., 0 = no suicidal ideation, 1 = suicidal ideation), in the autistic group, 23 (31.9%) women reported no suicidal ideation, compared to 49 (68.1%) who reported having suicidal ideation. In the non-autistic group, 295 (73.93%) women indicated no suicidal ideation, while 104 (26.07%) indicated having suicidal ideation. For the autistic group, the skewness was −0.79 (standard error = 0.28) and the kurtosis was −1.41 (standard error = 0.56), indicating a negatively skewed and relatively flat distribution, with responses clustering toward the higher end of the scale. In contrast, for the non-autistic group the skewness was 1.09 (standard error = 0.12) and the kurtosis was −0.81 (standard error = 0.24), indicating a positively skewed and relatively flat distribution, with most responses falling at the lower end of the scale.

Table 2 shows the mean (M) and standard deviation (SD) of the item 9 of the PHQ, GAD-7 total score, as well as the CAT-Q-ES – compensation, masking and assimilation subscales and total score –, for the autistic and non-autistic groups, as well as the t-test and the χ^2^ results. Overall, the autistic group scored statistically significantly higher on all dimensions, particularly large effects for anxiety and all CAT-Q-ES subscales (Cohen’s d = 0.82–1.58), indicating that autistic women report greater suicidal ideation, anxiety, and camouflaging strategies compared to non-autistic women (Table 2).

**Table 2:** Mean (M) and standard deviation (SD) values for the autistic and non-autistic women and groups differences in all variables.

Measures	Autistic		Non-autistic		χ^2^ (df) or t (df)	*p*	Cramer’s v or Cohen’s d	95% CI	
	Range	M(SD)	Range	M(SD)				Lower	Upper
PHQ-9	0–3	1.29 (1.16)	0–3	0.43	49.04 (1)	<0.001	0.32		
GAD-7 total score	0–21	14.50 (5.07)	0–21	9.38 (5.62)	−7.23 (469)	<0.001	−0.93	−1.18	−0.67
Compensation	12–63	47.24 (10.84)	9–60	27.45 (12.75)	−12.38 (469)	<0.001	−1.58	−1.85	−1.31
Masking	8–52	41.83 (6.63)	8–56	34.41 (9.42)	−6.40 (469)	<0.001	−0.82	−1.08	−0.56
Assimilation	15–56	45.74 (6.99)	8–56	31.29 (12.39)	−9.61 (469)	<0.001	−1.23	−1.49	−0.97
CAT-Q-ES total score	35–162	134.81 (19.47)	30–161	93.16 (30.72)	−11.10 (469)	<0.001	−1.42	−1.69	−1.25

### 3.2 Association of autism, anxiety, camouflaging, and age with suicidal ideation

A logistic regression analysis was conducted to test the associations between anxiety, camouflaging strategies (i.e., compensation, masking, and assimilation) and age with suicidal ideation, controlling for autism diagnosis and for having an anxiety and/or depression diagnosis. The model was significant, χ^2^ (8) = 192.64, *p* = <0.001, and explained 32% (McFadden R^2^ = 0.32) of the variability of suicidal ideation. When controlling for autism and having an anxiety and/or depression diagnosis, higher anxiety and depressive symptoms, as well as increased assimilation were statistically significantly associated with suicidal ideation. Autism diagnosis, anxiety and/or depression diagnosis, as well as compensation, masking and age were not significantly associated with suicidal ideation (Table 3).

**Table 3:** Unstandardized beta, z, and *p* values of the predictive variables of suicidal ideation.

Predictive variables	Coefficients				Collinearity values	
	Beta	SE	z	*p*	VIF	Tolerance
Depression diagnosis	1.03	0.35	2.92	<0.05	1.15	0.87
Anxiety diagnosis	0.06	0.31	0.19	0.85	1.24	0.81
Autism diagnosis	0.60	0.37	1.64	0.10	1.27	0.79
GAD-7 total score	0.15	0.03	5.65	<0.001	1.19	0.84
Compensation	−0.00	0.01	−0.22	0.83	2.52	0.40
Masking	−0.01	0.02	−0.47	0.64	1.88	0.53
Assimilation	0.07	0.02	4.55	<0.001	1.84	0.54
Age	−0.01	0.01	−1.30	0.19	1.04	0.96

SE = standard error; VIF = variance inflation factor.

### 3.3 Mediating role of anxiety in the relationship between camouflaging and suicidal ideation

**Figure 1:**
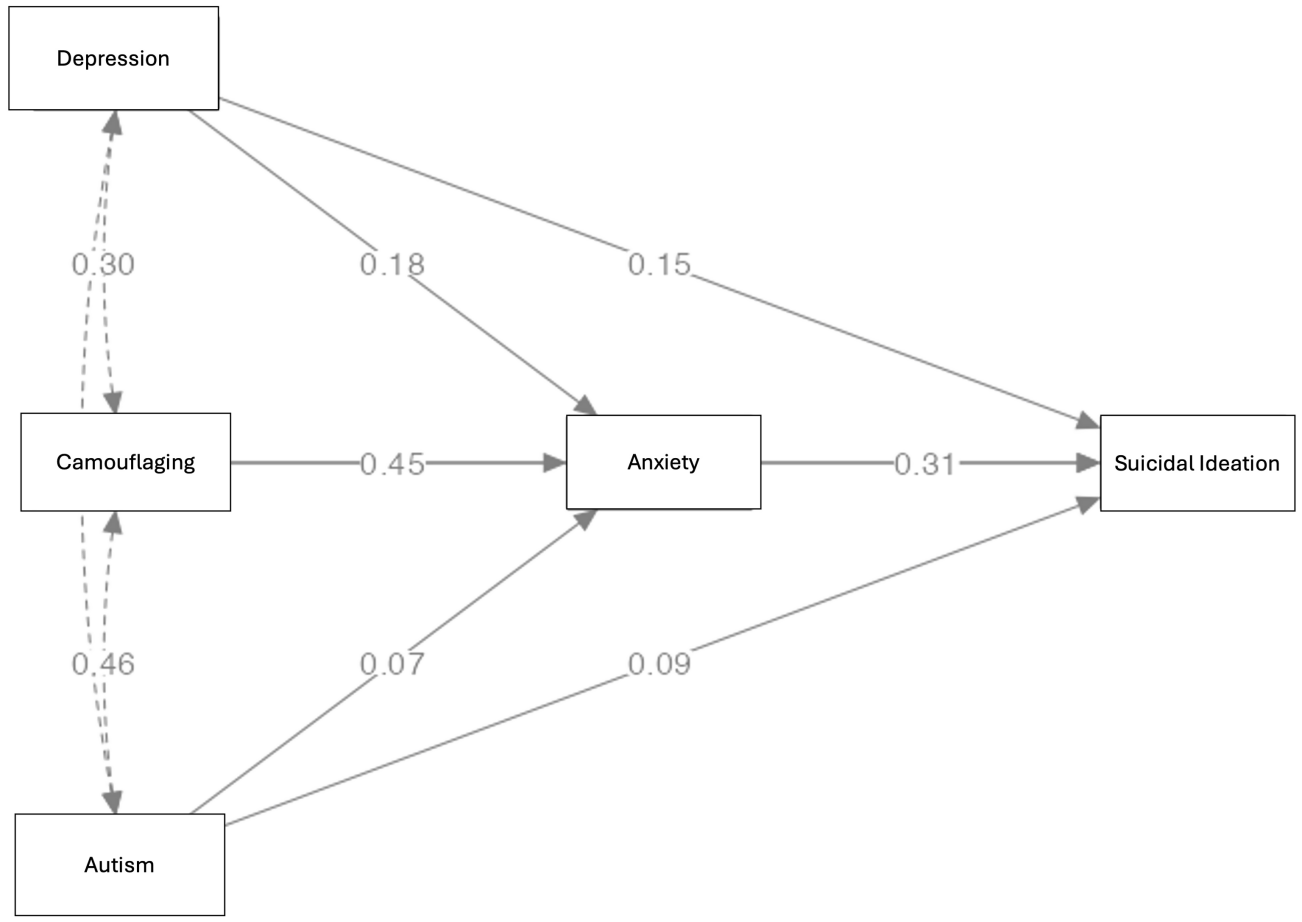
Mediation role of anxiety in the relationship between autism, camouflaging and suicidal ideation.

A mediation analysis was conducted to examine whether the statistical association between camouflaging and suicidal ideation was indirectly explained by anxiety, controlling for autism and depression diagnosis. The results are depicted in Figure 1 and Table 4. Positive and statistically significant indirect effects were observed, consistent with the hypothesis that anxiety accounted for part of the association between camouflaging and suicidal ideation (*β* = 0.14, *p* < 0.001). Specifically, we observed a positive and statistically significant association between anxiety and suicidal ideation (*β* = 0.31, *p* < 0.001), camouflaging and anxiety (*β* = 0.45, *p* < 0.001) and between depression diagnosis and anxiety (*β* = 0.18, *p* < 0.001). A statistically significant positive direct effect was found between autism and suicidal ideation (*β* = 0.10, *p* = 0.02) in the mediation analyses.

**Table 4:** The mediating role of anxiety in the relationship between camouflaging and suicidal ideation.

Effect	Estimate	SE	95% C. I.		*β*	z	*p*
			Lower	Upper			
(indirect) Autism → anxiety → suicidal ideation	0.03	0.02	0.01	0.06	0.02	1.63	0.10
(indirect) Camouflaging → anxiety → suicidal ideation	0.00	0.00	0.00	0.00	0.14	5.64	<0.001
(indirect) Depression diagnosis → anxiety → suicidal ideation	0.07	0.02	0.03	0.11	0.06	3.77	<0.001
Autism → anxiety	1.15	0.69	−0.20	2.50	0.07	1.67	0.09
Anxiety → suicidal ideation	0.02	0.00	0.02	0.03	0.31	6.71	<0.001
Camouflaging → anxiety	0.08	0.01	0.07	0.10	0.45	10.41	<0.001
Depression diagnosis anxiety	2.91	0.64	1.66	4.17	0.18	4.55	<0.001
(direct) Autism → suicidal ideation	0.12	0.06	0.01	0.23	0.09	2.22	0.03
(direct) Camouflaging → suicidal ideation	0.00	0.00	0.00	0.00	0.22	4.54	<0.001
(direct) Depression diagnosis → suicidal ideation	0.20	0.05	0.10	0.30	0.15	3.82	<0.001
(total) Autism → suicidal ideation	0.15	0.06	0.04	0.26	0.12	2.62	0.01
(total) Camouflaging → suicidal ideation	0.01	0.00	0.00	0.01	0.36	7.87	<0.001
(total) Depression diagnosis → suicidal ideation	0.27	0.05	0.17	0.38	0.21	5.07	<0.001

Models were analyzed using 95% bootstrap (percentiles) confidence intervals (CI). Betas are completely standardised effect sizes.

## 4 Discussion

Women are a particularly vulnerable group to mental health problems, and autistic women are at even greater risk, as camouflaging may be an additional burden on top of increased mental health problems such as anxiety and suicidal ideation. However, relationships between autism, anxiety, and camouflaging remain poorly studied, so their contribution to suicidal ideation in women is far from clear. Therefore, this study aimed (i) to examine the contribution of autism, camouflaging and anxiety to suicidal ideation, and (ii) to investigate the mediating role of anxiety in the relationship between camouflaging and suicidal ideation.

### 4.1 Group differences in camouflaging, anxiety and suicidal ideation

As expected, autistic women reported using more camouflaging strategies than non-autistic women. This is in line with other evidence showing that autistic adults or adults who report having high autistic traits use more camouflaging strategies, particularly women (Cook et al., 2021). Autistic women often use camouflaging as a coping mechanism to conceal their autistic traits to facilitate their adjustment to social environments and be accepted by peers (Tubío-Fungueiriño et al., 2021). Thus, our findings are consistent with other literature suggesting that autistic women tend to use more camouflaging to display behaviours that are considered more ‘appropriate’, perhaps to be accepted in social settings (Lai et al., 2017).

However, little evidence exists on how autistic and non-autistic women differ in using camouflaging. To the best of our knowledge, only one study has reported on the use of camouflaging comparing autistic and non-autistic women and no group differences were found (Hull et al., 2020). Thus, our findings contribute to the existing literature on camouflaging gender differences by showing that autistic women camouflage more compared to non-autistic women, but camouflaging differences in autistic and non-autistic women need further investigation. It is therefore important that future studies address this issue, as more evidence is needed to better understand possible camouflaging differences between neurodivergent and non-autistic women.

Similarly, autistic women reported more suicidal ideation as well as more anxiety and depression symptoms than non-autistic women. The present findings are consistent with previous evidence indicating that autistic people are at elevated risk for suicidal thoughts and behaviours compared to non-autistic people (Brown et al., 2024; Kirby et al., 2024; Newell et al., 2023) being autistic women at higher risk of internalizing disorders than autistic men (Green et al., 2019). The present findings also support the hypothesis that autistic women may be more vulnerable to internalising symptoms and psychiatric conditions due to their position as a doubly minoritised population (Grzeszak and Pisula, 2025). Several social factors may exacerbate this vulnerability, including adverse workplace experiences and job instability (Hayward et al., 2018), as well as various forms of victimization. These include bullying related to not conforming to gender norms, and relational and sexual violence, which have been associated with anxiety symptoms in autistic girls compared to boys (Cazalis et al., 2022; Greenlee et al., 2020). Other relevant social factors include internalised stigma for not conforming to social norms (Wilson et al., 2023), gender roles, feelings of being an “impostor” following diagnosis (Harmens et al., 2022) and camouflaging. The latter has been linked to an emotional burden, stigma, identity loss, and a lack of support (Jones et al., 2025). These experiences may contribute to an increase in internalising symptoms and then associated with a higher risk of suicidal ideation. Clinicians should explore the impact of social pressures, particularly those related to gender expectations and female stereotypes, on autistic women’s histories of victimisation and internalised stigma, and how these factors contribute to an increased risk of suicidal ideation.

### 4.2 Variables associated with suicidal ideation

As expected, our results showed that, when we consider the autism diagnosis and anxiety and depression diagnoses, anxiety and depressive symptoms and assimilation were associated with increased suicidal ideation. The present results suggest that suicidal ideation may be partly explained by internalising symptoms and the camouflaging strategy of assimilation. Once the co-occurrence of anxiety and depression conditions is controlled, the diagnosis of autism itself does not increase suicidal ideation.

In addition, higher anxiety levels were associated with increased suicidal ideation, which is in accordance with a large body of evidence suggesting that mental health problems, particularly anxiety, are strongly associated with increased suicidal thoughts or behaviours, making it a potential risk factor for suicidal ideation (Bentley et al., 2016; Nepon et al., 2010). The present results suggest that autistic and non-autistic women who tend to report higher internalising symptoms may be at greater risk for thinking about or planning to commit suicide.

Camouflaging was also associated with suicidal ideation, consistent with other evidence suggesting that camouflaging is linked to mental health outcomes (Beck et al., 2020; Cook et al., 2021; Somerville et al., 2024). However, our results showed that only assimilation strategies were associated with suicidal ideation. Assimilation refers to the adoption of observed attitudes and/or behaviours of others to fit into social environments (Hull et al., 2020), which may reflect women’s motivation to be accepted by others by meeting societal expectations (Hull et al., 2017). This may involve additional mental effort, placing greater psychological demands on women and being asssociated with increased suicidal ideation. Indeed, evidence shows that assimilation is associated with greater feelings of loneliness and emotional distress (Hull et al., 2017) and lower psychological well-being (Perry et al., 2022), which may be related to increased suicidal thoughts and behaviours. Some aspects of camouflaging behaviours are shared across neurotypes, and practitioners should consider underlying trauma and stigma experiences that may have led to the development of these social coping strategies in autistic and non-autistic women (Ai et al., 2022; Miller et al., 2021).

The lack of an additional effect of an autism diagnosis on suicidal ideation when considering anxiety and depressive disorders supports the evidence showing that women are a vulnerable group and at higher risk for internalizing symptoms, regardless of diagnosis (Asher et al., 2017; McLean et al., 2011). The literature suggests that several factors may contribute to women’s increased vulnerability to internalising disorders, including psychological factors, such as gender role expectations (i.e., femininity or female stereotypes), low self-esteem, problems in interpersonal relationships (Farhane-Medina et al., 2022), or increased exposure to social (e.g., discrimination), economic (e.g., poverty) and environmental (e.g., inequalities) stressors (Baños and Miragall, 2024; Seedat et al., 2009).

In addition, these findings emphasise the link between internalising symptoms and suicidal thoughts and behaviours, particularly among autistic women. Miranda-Mendizabal et al. (2019) identified depression as a risk factor for suicide attempts in women, particularly during adolescence and young adulthood. Doering et al. (2024) found that individuals with anxiety symptoms close to the time of suicide were more likely to be women, and that anxiety was more common among younger adults than older adults. As autistic women are at an increased risk of experiencing different types of victimisation and stigma than non-autistic women, these factors may potentially exacerbate anxiety and depressive symptoms, thereby contributing to the development of suicidal thoughts and behaviours more than an autism diagnosis itself.

Suicidal ideation has been proposed as an avoidance or decision-making bias to escape painful emotions (Millner et al., 2019). Therefore, our findings suggest that it is possible that women that have higher levels of anxiety and depression and use more assimilation strategies may experience greater psychological distress, and that suicidal ideation may be a potential avoidant strategy to cope with this distress. The present results highlight the need to address internalising symptoms, as well as socio-emotional motivations and coping strategies, in psychological interventions with women. However, it is necessary to determine the strength of this empirical information, and therefore future studies exploring these relationships are needed.

### 4.3 The mediating role of anxiety

Our results showed that anxiety helped explain part of the relationship between camouflaging and suicidal ideation. As expected, we found those who camouflage more tend to report increased anxiety which may, in this context, relate to greater suicidal ideation. Thus, this result suggests that the association between camouflaging and suicidal ideation may be partly explained by increased anxiety underlying this association, and this is particularly relevant for autistic women.

Research shows that camouflaging autistic traits is associated with increased anxiety in women, who often report that using such strategies is exhausting and stressful, as it requires significant cognitive and emotional resources (Hull et al., 2017; Tierney et al., 2016). These increased anxiety levels may be related to greater social isolation, reduced quality of life and altered functioning outcomes (Bentley et al., 2016). Such difficulties may contribute to autistic women feeling misunderstood or overlooked, which in turn has been associated with more suicidal thoughts and behaviours (Cage et al., 2018). This is consistent with other evidence suggesting that camouflaging may be an anxiety-related experience, associated with poorer mental health. For example, a mixed-methods systematic review of 4,808 autistic people (the majority of whom were white, female sex, and late-diagnosed autistic adults) found that camouflaging is related to social pressure from non-autistic people, the need for social acceptance and self-esteem (the need to fit in and belong), but at a great cost to their well-being, including increased anxiety (Zhuang et al., 2023). Accordingly, camouflaging appears to be associated with increased feelings of anxiety, which may thereby be linked to feelings of being overlooked, unsupported and burnout, negatively impacting on social relationships and identity, and potentially contributing to increased suicidal ideation.

Notably, anxiety also accounted for part of the relationship between a depression diagnosis and suicidal ideation, but not between autism diagnosis and suicidal ideation. The finding that anxiety levels partly explain the risk of suicidal ideation in people with a depression diagnosis is consistent with the high co-occurrence of anxiety and depression disorders, as well as their combined effect on suicidality (Sanches et al., 2022). The present study suggests that suicidal ideation in autistic women may not be directly related to their diagnosis, but rather to the high prevalence of anxiety and depressive symptoms and diagnoses. These results indicate a complex interplay of factors. Being a woman is associated with a higher risk of anxiety and depression, both of which are independently linked to suicidal ideation. In particular, autistic women are at greater risk of victimisation, which can lead to a range of internalising symptoms and elevated suicidal ideation compared to non-autistic women. Finally, camouflaging, which is associated with stigma and an emotional burden, appears to be relevant for both autistic and non-autistic women. Overall, these findings highlight the importance of considering potential factors underlying camouflaging in autistic women. Importantly, it shows that anxiety may be an important aspect to consider in the relationship between camouflaging and suicidal ideation and to target in gender-sensitive psychological intervention approaches.

### 4.4 Limitations and future studies

This study has some limitations. Firstly, our sample is imbalanced in terms of autism diagnosis status because the group of autistic women was smaller than the non-autistic group. Therefore, to replicate these results, future studies should examine the link between autism diagnosis, camouflaging and suicidal ideation in a larger and heterogeneous group of participants. Additionally, participants were recruited and invited to take part in this study online. Online studies may be biased toward people with a particular interest in the topic (such as women with late-diagnosed autism), higher cognitive ability and higher levels of education. Bjelland et al. (2008) reported that adults aged 20 years and above with a higher educational level experienced fewer symptoms of anxiety and depression, suggesting an accumulating protective effect of education throughout life. However, a recent systematic review and meta-analysis (Chi et al., 2023) highlighted a high risk of anxiety disorder among postgraduate students without significant differences between pre- and post-COVID. Although age was not significantly associated with suicidal ideation in this study, evidence suggests an interaction between age, education, and anxiety that should be considered in future studies. Therefore, the generalisation of these results to women with lower educational levels or intellectual disabilities is limited. Although the PHQ-9 item 9 may indicate suicidal ideation in autistic and non-autistic groups, relying solely on this item has several limitations. Firstly, it has not been specifically validated in autistic adults. Secondly, it conflates passive suicidal ideation with risk of self-harm. Thirdly, it only considers frequency, leaving aspects such as intensity, planning, and impulse control unassessed. Furthermore, Na et al. (2018) observed that the PHQ-9 suicide item exhibited poor validity when compared to the gold standard definition of suicidal ideation, and that it must be used in conjunction with a suicide-specific measure. Another limitation of this work is that it does not include overall depressive symptoms, due to their substantial overlap with the suicidal ideation measure. In addition, this study only considered symptoms of generalised anxiety and did not measure symptoms of other anxiety disorders, such as social anxiety, which has also been linked to camouflaging (Hull et al., 2021). Future studies should assess depressive symptoms and other internalising disorders (e.g., social anxiety) with standardised measures to clarify their role in the relationship between camouflaging and suicidal ideation. In view of the absence of suicide-specific measures that have been validated in Spanish autistic individuals, the development of new measures would be a significant step toward a better understanding suicidality in Spanish autistic women.

### 4.5 Conclusion

This study highlights the complex interplay between autism, camouflaging, anxiety, and suicidal ideation in women and reinforces the importance of considering anxiety as a mechanism contributing to the association between camouflaging and suicidal ideation, especially in autistic women. Additionally, the present work suggests that a greater risk of co-occurring internalising disorders explained suicidal ideation better than autism diagnosis itself in women. However, more research is needed to clearly understand possible factors underlying suicidal ideation in this group, particularly in autistic women. A clear understanding of these factors is crucial for the design of sensitive and targeted interventions and preventive approaches. Although our study highlights the important relationships between camouflaging, anxiety and suicidal ideation in autistic women. Studies of gender differences must help to understand and address the increased risk of suicidal ideation, behaviour and death in autistic and non-autistic people across genders.

### 4.6 Community involvement

The recruitment process for this study was supported by the dissemination of the survey through autism associations, institutions, federations, clinical units, autism-related research networks, and social media. Additionally, autistic individuals contributed to the cultural adaptation of the Camouflaging Autistic Traits Questionnaire (CAT-Q-ES) to the Spanish context, ensuring its relevance and appropriateness for the target population.

## Data Availability

The datasets presented in this article are not readily available due to privacy and ethical restrictions. Requests to access the datasets should be directed to montse.fernandez.prieto@usc.es.
